# Candidate biomarkers of EV-microRNA in detecting REM sleep behavior disorder and Parkinson’s disease

**DOI:** 10.1038/s41531-023-00628-4

**Published:** 2024-01-10

**Authors:** Yuanyuan Li, Ying Cao, Wei Liu, Fangzheng Chen, Hongdao Zhang, Haisheng Zhou, Aonan Zhao, Ningdi Luo, Jun Liu, Ligang Wu

**Affiliations:** 1 Department of Neurology & Institute of Neurology, Ruijin Hospital affiliated to Shanghai Jiaotong University School of Medicine, Shanghai, China; 2 Key Laboratory of RNA Science and Engineering, CAS Center for Excellence in Molecular Cell Science, Shanghai Institute of Biochemistry and Cell Biology, Chinese Academy of Sciences, University of Chinese Academy of Sciences, Shanghai, China; 3 MOE Key Laboratory of Bioinformatics, Center for Synthetic and Systems Biology, School of Life Sciences, Tsinghua University, Beijing, China; 4 Institute for Precision Medicine, Tsinghua University, Beijing, China; 5 Lingang Laboratory, Shanghai, China

**Keywords:** Parkinson's disease, Diagnostic markers

## Abstract

Parkinson’s disease (PD) lacks reliable, non-invasive biomarker tests for early intervention and management. Thus, a minimally invasive test for the early detection and monitoring of PD and REM sleep behavior disorder (iRBD) is a highly unmet need for developing drugs and planning patient care. Extracellular vehicles (EVs) are found in a wide variety of biofluids, including plasma. EV-mediated functional transfer of microRNAs (miRNAs) may be viable candidates as biomarkers for PD and iRBD. Next-generation sequencing (NGS) of EV-derived small RNAs was performed in 60 normal controls, 56 iRBD patients and 53 PD patients to profile small non-coding RNAs (sncRNAs). Moreover, prospective follow-up was performed for these 56 iRBD patients for an average of 3.3 years. Full-scale miRNA profiles of plasma EVs were evaluated by machine-learning methods. After optimizing the library construction method for low RNA inputs (named EVsmall-seq), we built a machine learning algorithm that identified diagnostic miRNA signatures for distinguishing iRBD patients (AUC 0.969) and PD patients (AUC 0.916) from healthy individuals; and PD patients (AUC 0.929) from iRBD patients. We illustrated all the possible expression patterns across healthy-iRBD-PD hierarchy. We also showed 20 examples of miRNAs with consistently increasing or decreasing expression levels from controls to iRBD to PD. In addition, four miRNAs were found to be correlated with iRBD conversion. Distinct characteristics of the miRNA profiles among normal, iRBD and PD samples were discovered, which provides a panel of promising biomarkers for the identification of PD patients and those in the prodromal stage iRBD.

## Introduction

Parkinson’s disease (PD) is the second most common neurodegenerative disease, imposing increasingly heavy social and economic burdens on aging societies^1,2^. Idiopathic rapid eye movement (REM) sleep behavior disorder (iRBD) is a parasomnia characterized by the loss of normal atonia during the REM stage of sleep which results in overt motor behaviors that frequently represent the enactment of dreams^3^. In the past decade, iRBD has been established as one of the earliest and most specific prodromal signs of α-synucleinopathies, including PD, dementia with Lewy bodies (DLB) and multiple system atrophy (MSA)^3^. Thus, accessible and reliable biomarkers for early diagnosis of PD and iRBD are urgently needed to identify candidate therapeutic targets and to monitor disease progression during therapeutic interventions^4,5^.

Currently, the diagnosis of PD mostly relies on clinical symptoms, which hampers the detection of the earliest phases of the disease, the time at which treatment may have the greatest therapeutic effect. A variety of biomarkers for diagnosing PD are under investigation, including factors based on pathological, imaging, biochemical, and genetic data^4^. Biofluid biomarkers have markedly expanded over the past 5 years, including α-synuclein, lysosomal enzymes, and neurofilament light chain in CSF^6,7^. Blood biomarkers, such as α-synuclein, are also under investigation; whereas their quantities are strongly influenced by red blood cell (RBC) contamination and haemolysis, which limit the utility for diagnostic purposes^8^. Besides, although non-invasive neuroimaging techniques can provide in-depth information about brain structure and function, these methods require major investments in infrastructure which limit their wide clinical deployment^9^. Therefore, a minimally invasive test for early detection of iRBD and PD and for monitoring disease progression still poses a major challenge.

In consideration of potential biomarkers, microRNAs (miRNAs) are small non-coding RNAs (sncRNAs) that are generally expressed in all eukaryotic cells, and which perform critical regulatory functions at the posttranscriptional level. Cellular miRNAs released into body fluids can be readily detected, making them ideal biomarkers for diagnosis of various diseases^10^. Previous studies have reported that salivary miR-153 and miR-223 can be used as biomarkers for idiopathic PD^11^, while serum miR-221 is a potential predictor of PD^12^. In addition, circulating brain-enriched miRNAs have been used to distinguish idiopathic and genetic PD^13^. However, all of these studies used reverse transcription followed by real-time quantitative PCR (RT-qPCR) method to measure the relative expression of a few miRNAs in bodily fluids. Since this method detects a limited number of known miRNAs and is also inefficient in distinguishing miRNAs with similar sequences^14^, the most diagnostically informative miRNAs could be potentially overlooked.

Extracellular vesicles (EV) are nano-scale, membrane-enclosed particles released from (possibly) all eukaryotic cells to transport proteins, lipids, RNAs, and DNA fragments^15^, a process shown to have important biological functions^16,17^. EVs have been found in serum, plasma, urine, saliva, cerebrospinal fluid, and breast milk^15^. Moreover, the EV membrane effectively prevents degradation of the enclosed miRNAs by ribonucleases that are abundant in biofluids^18,19^. These features of EV-associated miRNAs cumulatively enhance miRNA biomarker reliability compared with unprotected, cell-free, miRNAs^20–23^. However, the full spectrum of EV-associated miRNAs presents in the plasma of iRBD and PD patients remains unknown, and characterizing the miRNA population in PD or iRBD patients may unveil several effective diagnostic biomarkers. In this study, we used high throughput sequencing to profile the EV-associated sncRNA population in the plasma of iRBD and PD patients. Here, we report several such candidate EV-miRNA biomarkers for diagnosis of iRBD and PD patients.

## Results

### Clinical characteristics of participants

A total of 169 participants were enrolled in this study and subsequently divided into three groups consisting of 56 iRBD patients, 53 PD patients, and 60 healthy individuals. The demographics and clinical characteristics of the enrolled participants are listed in Table 1. No significant differences were found in age distribution among the three groups, although the PD group contained a significantly higher proportion of female subjects. The average disease duration for iRBD patients was 7 years, and 5 years for PD patients. In addition, the iRBD patient group exhibited a higher average RBDSQ score than that of the PD group (*P* < 0.001), while PD patients showed lower SS-16 (*P* = 0.03) and higher SCOPA-AUT (*P* = 0.001) scores, on average, than iRBD patients. The HAMD scores were higher in PD patients than in healthy controls (*P* < 0.001). No significant differences in NMSQ and HAMD scores were observed between the iRBD and PD groups. Models were then trained on a training set (60% of data) and evaluated on a validation set (remaining 40% of the data), with an equal distribution of individuals in the disease groups (Supplementary Table 1).

**Table 1:** Demographic and clinical profiles of healthy, iRBD, and PD participants.

Clinical parameters	Healthy	iRBD	PD	*P* value
No. of participants	60	56	53	/
Age	63.5 ± 9.0	64.0 ± 7.3	63.0 ± 9.0	0.62
Sex (M/F)	35/25	34/22	25/28	<0.001
Disease duration (y)	/	7.0 ± 5.1	6.9 ± 12.4	/
UPDRS III	/	/	30.6 ± 18.0	/
RBDSQ	/	8.1 ± 3.2	4.4 ± 3.2	<0.001
NMSQ	/	7.0 ± 3.9	8.2 ± 3.4	0.09
SS-16	/	8.8 ± 4.4	7.1 ± 3.5	0.03
HAMD	/	3.6 ± 4.0	5.1 ± 5.0	0.11
SCOPA-AUT	/	7.8 ± 4.7	14.4 ± 12.9	0.001

*iRBD* idiopathic rapid eye movement sleep behavior disorder, *PD* Parkinson’s disease, *UPDRS III* the Unified PD Rating Scale III, *RBDSQ* the REM Sleep Behavior Disorder Screening Questionnaire, *NMSQ* Non-Motor Symptom Questionnaire, *SS-16* the Sniffin’ Sticks 16-item test, *HAMD* the 17-item Hamilton Depression Rating Scale, *SCOPA-AUT* Scale for Outcomes in PD-Autonomic.

### Identification of PD-specific miRNA biomarkers

**Fig. 1:**
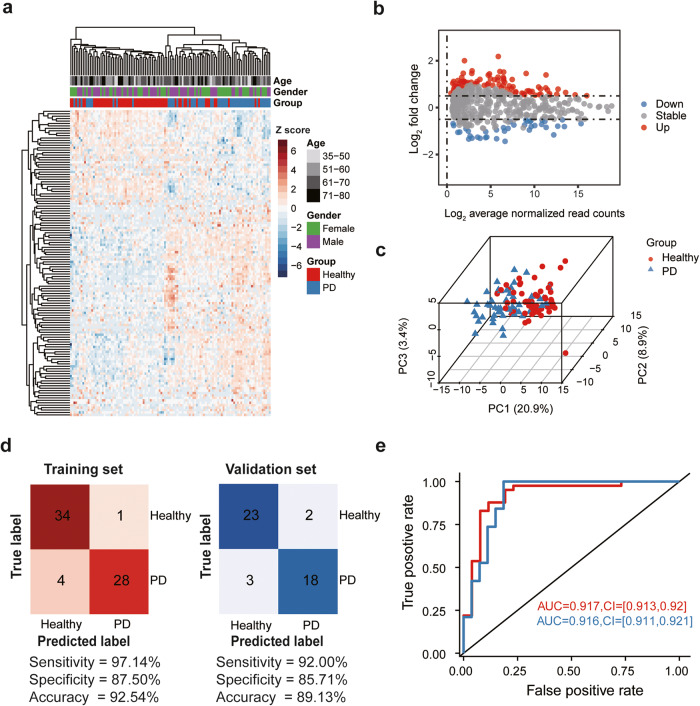
EV-associated miRNA biomarkers for detecting PD. **a** Unsupervised clustering heatmap of differentially expressed miRNAs for PD patients (blue) and healthy individuals (red). Each row represents a differentially expressed miRNA. **b** MA-plot of differentially expressed miRNAs between PD patients and healthy individuals by transforming read abundance onto M (log_2_ fold change) and A (log_2_ average normalized read counts) scales. Up- or down-regulated miRNAs are shown in red or blue, respectively. **c** Three-dimensional scatter plot of principal component analysis (PCA) for the differentially expressed miRNAs between PD patients and healthy individuals. The percentage of variance explained by PC1, PC2, and PC3 are shown in the label. **d** Confusion matrix of the support vector machine (SVM) classifier for PD patients and healthy individuals in the training set (left) and validation set (right). The sensitivity, specificity, and accuracy are shown at the bottom. **e** The receiver operating characteristic (ROC) curve of the SVM classifier in the training set (Red, *n* = 67) and validation set (blue, *n* = 46). The values of area under the curve (AUC) and 95% confidence interval (CI) are shown in the plot.

We next sought to determine whether plasma EV-associated miRNA profiles could be used to distinguish PD patients from healthy individuals. We identified a total of 87 upregulated and 39 downregulated miRNAs unique to the PD patient group (Fig. 1a, b, Supplementary Data 1). Unsupervised hierarchical clustering and principal component analysis (PCA) of the differentially expressed miRNAs separated PD patient samples from healthy controls with minor overlap (Fig. 1c). We then constructed a support vector machine (SVM) classifier to distinguish PD patients. Feature selection from the training set (n = 67) identified 15 informative miRNAs (Supplementary Data 2), including 10 upregulated miRNAs (miR-27b-3p, miR-199a-5p, miR-151a-3p, miR-584-5p, miR-889-3p, miR-619-5p, miR-130b-5p, miR-197-3p, miR-4433b-5p, and miR-4433a-3p) and 5 downregulated miRNAs (miR-96-5p, miR-155-5p, miR-150-5p, miR-150-3p, and miR-3615). We then used a 5-fold cross-validation SVM algorithm to classify PD patients from healthy individuals, which showed 97.14% sensitivity, 87.5% specificity, and 92.54% accuracy in the training set (35 healthy controls and 32 patients with PD) and 92% sensitivity, 85.71% specificity and 89.13% accuracy in the validation data set (25 healthy controls and 21 patients with PD) (Fig. 1d). The classifier could also identify patients with PD from healthy individuals in both the training (AUC = 0.917; 95% CI, 0.913-0.92) and validation (AUC = 0.916; 95% CI, 0.911-0.921) sets (Fig. 1e). These results suggested that the 15 miRNA features from the sncRNA profile of PD patients could be used to distinguish PD samples from healthy controls.

### Identification of iRBD-specific miRNA biomarkers

**Fig. 2:**
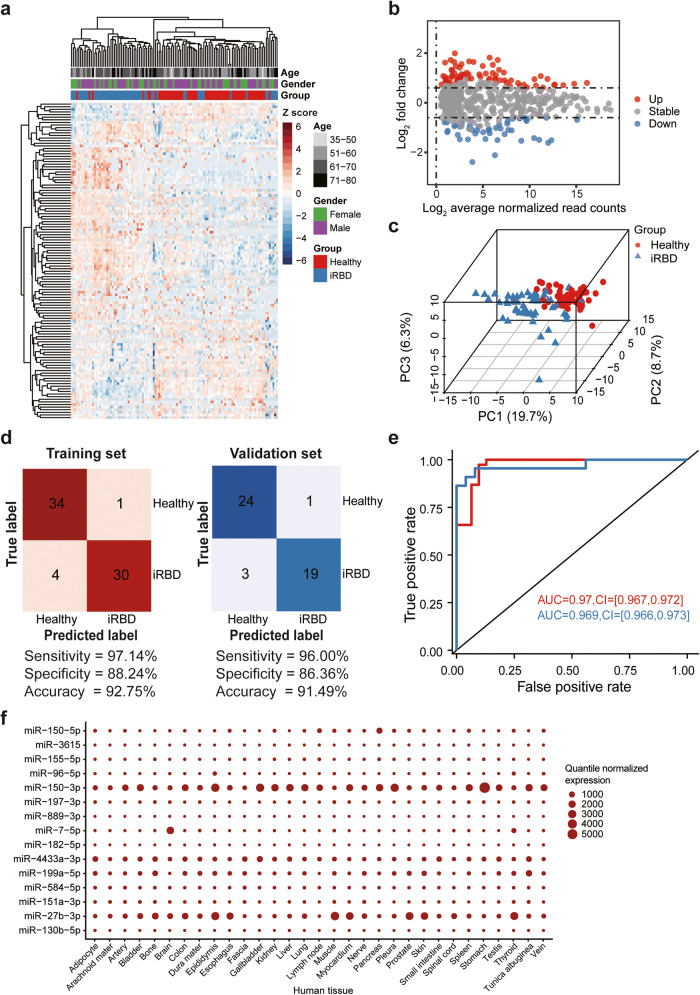
EV-associated miRNA biomarkers for detecting iRBD. **a** Unsupervised clustering heatmap of differentially expressed miRNAs of iRBD patients and healthy individuals (red). Each row represents a differentially expressed miRNA. **b** MA-plot of the differentially expressed miRNAs between iRBD patients and healthy individuals. The up- or down-regulated miRNAs are shown in red or blue, respectively. **c** Three-dimensional scatter plot of PCA for the differentially expressed miRNAs between iRBD patients and healthy individuals. **d** Confusion matrix of the SVM classifier for iRBD patients and healthy individuals in the training set (left) and validation set (right). The sensitivity, specificity, and accuracy are shown at the bottom. **e** The ROC curve of the SVM classifier in training set (red, *n* = 69) and validation set (blue, *n* = 47). The values of AUC and 95% confidence interval are shown in the plot. **f** The expression levels of PD- and iRBD-specific miRNA biomarkers in human tissues.

To identify patients in this early stage of pathogenesis, we further explored whether EV miRNAs could distinguish iRBD patients from healthy individuals. A total of 75 differentially upregulated and 46 downregulated miRNAs were identified among the total plasma EV-associated sncRNAs expressed in iRBD patients compared with healthy subjects (Fig. 2a, b, Supplementary Data 3). Unsupervised hierarchical clustering and PCA based on the differentially detected miRNAs separated iRBD patient samples from control samples with minor overlap (Fig. 2c). We performed feature selection (*n* = 69) and resulted in a miRNA signature comprised of three informative features. Specifically, miR-27b-3p was upregulated in the iRBD group, while miR-182-5p and miR-7-5p were downregulated (Supplementary Data 4). The diagnostic performance of this training set showed 97.14% sensitivity, 88.24% specificity, and 92.75% accuracy and independent validation of the training model with a separate validation set (*n* = 47) revealed 96% sensitivity, 86.36% specificity, and 91.49% accuracy (Fig. 2d). Ultimately, this classifier could effectively distinguish iRBD patients from control subjects in both the training (AUC 0.97; 95% CI, 0.967–0.972) and validation (AUC 0.969; 95% CI, 0.966–0.973) datasets (Fig. 2e), which indicated that the three miRNA signature features could sufficiently distinguish iRBD patients. Moreover, we found that several iRBD or PD related EV-associated miRNAs were highly expressed in the human brain, including miR-7-5p and miR-27b-3p (Fig. 2f), which may be secreted as EV-associated miRNAs by neuronal cells in the brain.

### Identification of miRNAs for distinguishing iRBD and PD

**Fig. 3:**
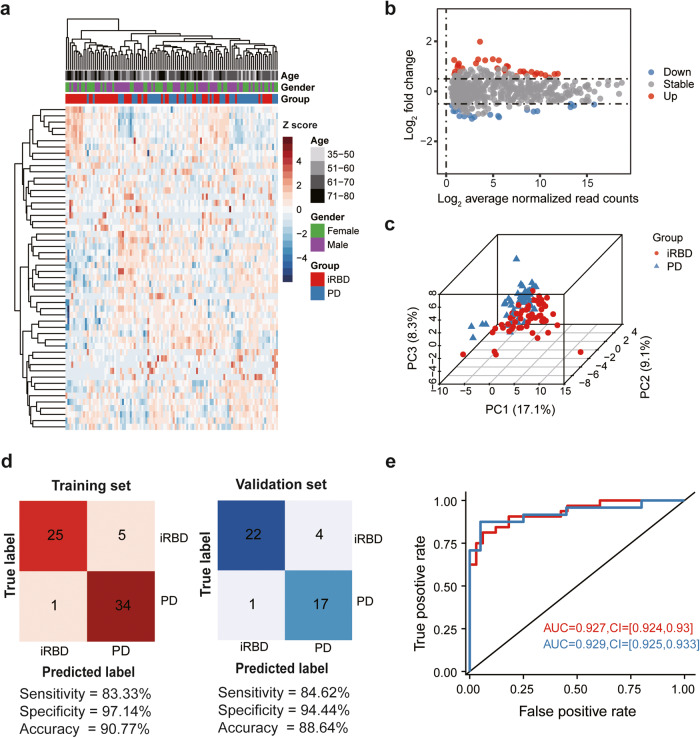
EV-associated miRNAs for distinguishing iRBD and PD. **a** Unsupervised clustering heatmap of differentially expressed miRNAs in PD patients (blue) and iRBD patients (red). Each row represents a differentially expressed miRNA. **b** MA-plot of the differentially expressed miRNAs between PD patients and iRBD patients. The up- or down-regulated miRNAs are shown in red or blue, respectively. **c** Three-dimensional scatter plot of PCA for the differentially expressed miRNAs between PD patients and iRBD patients. **d** Confusion matrix of the SVM classifier for PD patients and iRBD patients in the training (left) and validation (right) sets. Sensitivity, specificity, and accuracy are shown at the bottom. **e** ROC curve of the SVM classifier in the training (red, *n* = 65) and validation (blue, *n* = 44) sets. AUC values and 95% confidence intervals are shown in the plot.

Moreover, we further explored differences in miRNA levels between iRBD patients and PD patients and identified a total of 33 and 19 differentially up- and downregulated miRNAs, respectively, in total plasma EV-associated sncRNAs expressed in the latter group (Fig. 3a, b, Supplementary Data 5). Unsupervised hierarchical clustering and PCA based on the differentially detected miRNAs also separated PD from iRBD patient samples with minor overlap (Fig. 3c). We then identified a total of 9 miRNA in the training set, including 5 upregulated (miR-182-5p, miR-183-5p, miR-335-5p, miR-505-5p, and miR-340-3p) and 4 downregulated (miR-847-3p, miR-181b-5p, miR-4676-3p, and miR-2115-3p) miRNAs (Supplementary Data 6). The sensitivity, specificity, and accuracy of the SVM model built with these 9 miRNAs in the training set (*n* = 65) were 83.33%, 97.14%, and 90.77%, respectively, compared to 84.62%, 94.44%, and 88.64% in the validation set (*n* = 44) (Fig. 3d). The AUC values of the classifier to distinguish PD from iRBD patients in the training and validation sets were 0.927 (95% CI, 0.924–0.93) and 0.929 (95% CI, 0.925–0.933), respectively (Fig. 3e).

Furthermore, of the 53 PD patients studied, 34% (18/53) also presented with iRBD (i.e., PD-iRBD patients), a ratio similar to that reported in previous studies^24,25^. We further investigated the miRNAs discriminating PD-iRBD from iRBD patients by comparing their small RNA profiles. In total, we identified 31 up- and 13 downregulated miRNAs, with 480 miRNAs with no expression level changes between the groups (Supplementary Fig. 1a, b, Supplementary Data 7). Moreover, we found 20 differentially expressed miRNAs between PD patients without iRBD (i.e., PD-no-iRBD patients) and PD-iRBD patients (Supplementary Fig. 1c, d, Supplementary Data 8). Intriguingly, among the 20 differentially expressed miRNAs, 5 (miR-10a-5p, miR-874-3p, miR-4485-3p, miR-369-5p, and miR-625-5p) showed no significant differences in expression levels between iRBD patients and PD-iRBD patients but could distinguish PD-no-iRBD from both iRBD and PD-iRBD patients (Supplementary Fig. 1e).

### Longitudinal evaluation of candidate EV-miRNAs for conversion of iRBD

**Fig. 4:**
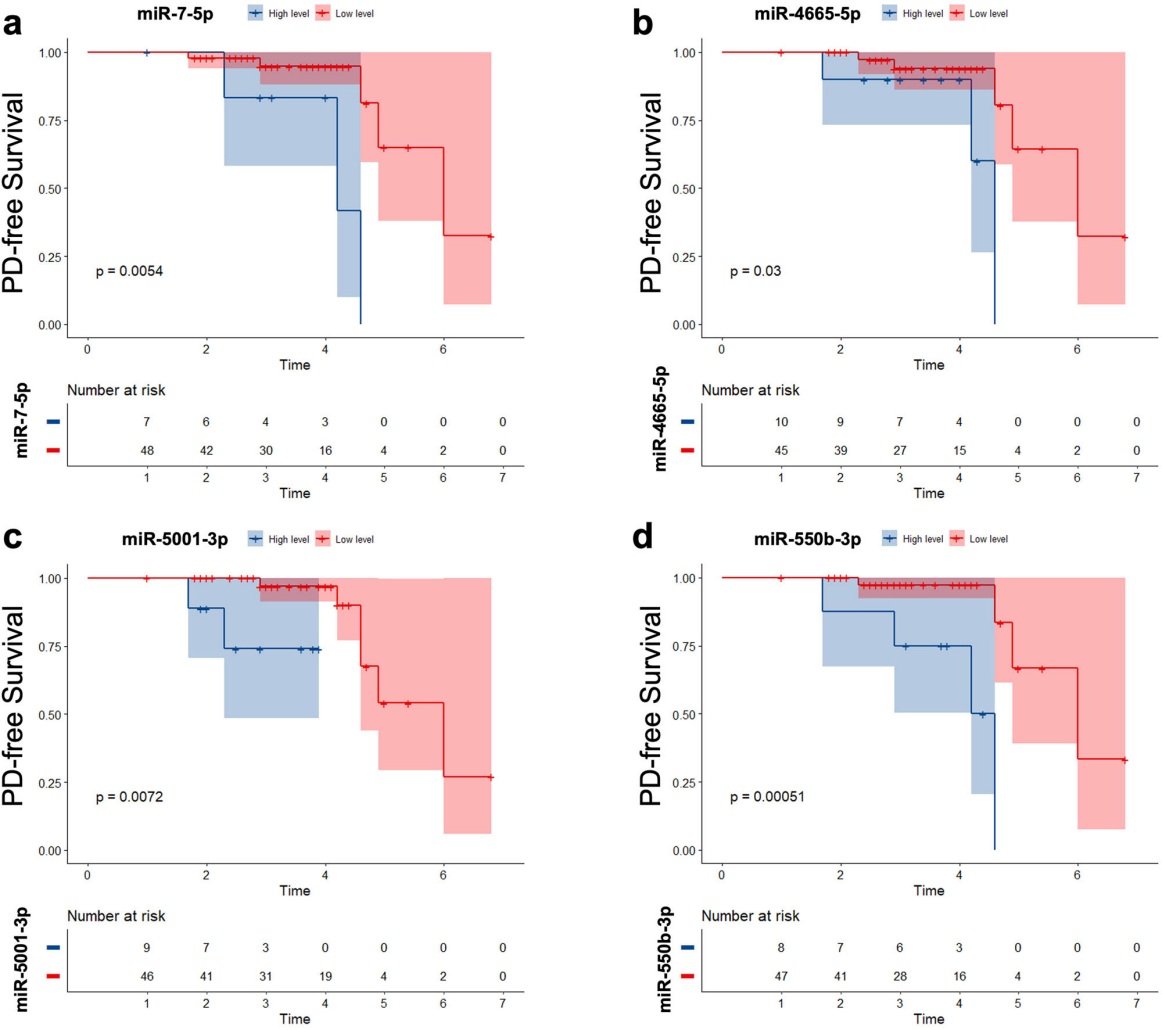
Longitudinal evaluation of candidate EV-miRNAs for conversion of iRBD. Kaplan-Meier plot of disease-free survival of patients with idiopathic REM sleep behavior disorder (iRBD), stratified according to EV-miRNA levels. **a** EV miR-7-5p; **b** EV miR-4665-5p; **c** EV miR-5001-3p; **d** EV miR-550b-3p. The solid line indicates patients with less or equal relevant levels.

In the longitudinal data, we found 8 iRBD patients converted to PD (7 patients)/MSA (1 patient) to the end of follow-up of 3.3 years. After adjustment for age and sex, plasma EV-derived miR-7-5p, miR-4665-5p, miR-5001-3p and miR-550b-3p were found to be correlated with RBD conversion (Supplementary Table 2). We divided the patients into groups according to plasma EV-derived miRNAs levels according to best cut-off values produced by R survival and survminer packages. Kaplan-Meier plots suggested that RBD patients with higher levels of plasma EV-derived miR-7-5p, miR-4665-5p, miR-5001-3p and miR-550b-3p were prone to convert to PD or MSA (Fig. 4).

### Identification of different miRNA expression patterns in the healthy-iRBD-PD hierarchy

**Fig. 5:**
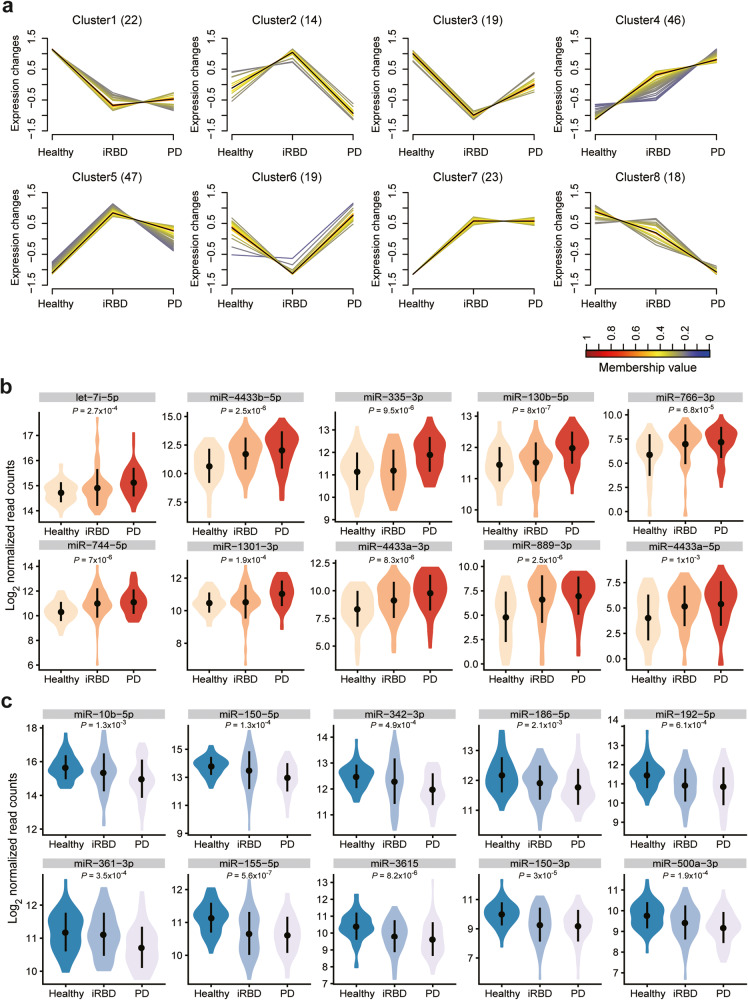
Identification of different miRNA expression patterns from healthy controls to iRBD to PD samples. **a** An expression pattern composed of 8 different miRNAs were identified in healthy controls, iRBD and PD groups. The number of miRNA species in the clusters is indicated in the figure. Each line represents a specific miRNA. The black line represents the cluster center. The membership values of miRNAs and the cluster center are indicated by the color gradient. Examples of 10 miRNAs with an expression that progressively increases (**b**) or decreases (**c**) from healthy controls to iRBD to PD samples. The p-values of a Kruskal-Wallis rank sum test for comparisons among the three groups are shown in the boxplot for each miRNA. The mean and standard deviation of miRNA expression levels are shown in black.

To next investigate whether (and which of) these miRNAs might be linked to PD development through iRBD, we compared the miRNA profiles in the plasma EVs derived from healthy, iRBD, and PD samples. A total of 208 miRNAs (Kruskal-Wallis *P* values < 0.05) were clustered into 8 significant discrete clusters (Fig. 5a, Supplementary Data 9). There were 46 miRNAs (Cluster4) progressively increased from healthy control to iRBD to PD samples and 40 miRNAs (Cluster1, Cluster8) progressively decreased from healthy control to iRBD to PD samples. We showed 20 miRNAs examples that consistently increased or decreased in expression from control to iRBD to PD patients (Fig. 5b, c). Let-7i-5p, miR-4433b-5p, miR-335-3p, miR-130b-5p, miR-766-3p, miR-744-5p, miR-1301-3p, miR-4433a-3p, miR-889-3p, and miR-4433a-5p all significantly increased in transcript abundance following the control<iRBD<PD hierarchy. By contrast, miR-10b-5p, miR-150-5p, miR-342-3p, miR-186-5p, miR-192-5p, miR-361-3p, miR-155-5p, miR-3615, miR-150-3p, and miR-500a-3p all decreased in expression following the order of control>iRBD>PD (Fig. 5c).

### Profiling EV-associated sncRNAs in plasma samples

Profiling the relative abundance of specific sncRNAs by high throughput sequencing typically requires tens of nanograms of total RNA^26,27^ and our initial attempts to construct a high-quality EV-associated sncRNA library using commercially available kits were unsuccessful, mainly due to the effects of low RNA inputs that result in 5’ and 3’ adapters generating the predominant ligation byproducts which severely compromise subsequent PCR amplification and sequencing quality. We, therefore, optimized the conditions for 5’ and 3’ adapter ligation and introduced an extra 5’ exonuclease treatment step that effectively reduced adapter dimer formation (Supplementary Fig. 2a). The EV RNAs (0.5 to 2 ng) treated with Cas9/sgRNA showed comparable and clear miRNA bands, indicating that we were able to reduce the input of EV RNA to 1 ng or less (Supplementary Fig. 2b). The Cas9/sgRNA in vitro cleavage step led to an effective reduction of both ysRNAs and adapter dimer byproducts, enabled a straightforward PAGE-based method for detection and recovery of miRNA products (~140 bp) (Supplementary Fig. 2c, d). Although the Cas9/sgRNA treatment couldn’t remove ysRNAs completely, the cleavage efficiency could be further increased by raising the Cas9/sgRNA concentration in the reaction. The comparison of sequencing results between the pairwise samples with or without Cas9/sgRNA treatment showed high correlations among miRNA profiles (Spearman correlation coefficient ≥ 0.99) (Supplementary Fig. 2e, f), which indicated that Cas9/sgRNA treatment had no obvious effect on the individual miRNA abundance.

## Discussion

Accessible and reliable biomarkers for early diagnosis of PD and iRBD are urgently needed to identify candidate therapeutic targets and to monitor disease progression during therapeutic interventions^5^. In this study, we investigated the expression of sncRNAs associated with plasma-derived EVs from 169 individuals using an improved library preparation protocol for high throughput sequencing. Our analysis revealed 15 EV-associated miRNA features that were diagnostically informative for PD, and three miRNA signature features that could serve as biomarkers for iRBD. Moreover, four EV- miRNAs associated with conversion of RBD to synucleinopathies.

The high-quality high-throughput sequencing data of sncRNAs in EVs were scarce, making it difficult to obtain sufficient useful information for the identification of specific biomarkers. We optimized the protocol for EV-sncRNA library construction and used Cas9/sgRNA system to remove the highly abundant endogenous-sncRNAs that had little correlation with diseases. Through these modifications, we successfully reduced the total required EV-RNA inputs to 0.5 ng. We named this optimized library construction method for sncRNA sequencing with low EV-RNA inputs as EVsmall-seq, which is suitable for clinical application and helpful to readers. Each library was sequenced to a mean depth of 19 million reads (median depth: 15 million) (Supplementary Fig. 3a, Supplementary Data 10). After low-quality and short reads being discarded, the average proportion of high-quality reads in these samples was 82.3% (Supplementary Fig. 3b), and 65.2% of these high-quality reads could be mapped to the reference sequence for sncRNAs indicating the overall high quality of the sncRNA libraries (Supplementary Fig. 3c). We found that the miRNAs (17-24 nt), ysRNAs (25–33 nt), tRNA-derived small non-coding (tsRNAs, 30–33 nt), and rRNA-derived small non-coding (rsRNAs, 17–20 nt) were the most prevalent types of sncRNAs associated with EVs (Supplementary Fig. 3d, e). On average, 390 different miRNA species were detected in each sample (average normalized read count >1, expressed frequency of all samples > 25%) (Supplementary Fig. 3f, Supplementary Data 11).

According to previous studies, some of these miRNAs may affect PD development by regulating PD-related genes. For example, miR-7-5p is expressed mainly in neurons and represses α-synuclein protein levels through targeting the 3’UTR of α-synuclein mRNA^28^. We also found a panel of EV-associated miRNAs that consistently increased or decreased in expression levels when comparing healthy subjects to iRBD and PD patients, which might be linked to iRBD-mediated PD development. In support of this hypothesis, it has been previously reported that miR-150-5p expression is dysregulated during PD progression^29^. In addition, previous studies conducted on let-7 showed its involvement in the regulation of neuronal degeneration in PD^30^ and promoting inflammation and neuronal death^31^. Moreover, the accumulation of let-7 can lead to increased α-synuclein expression and decreased autophagy^32^. Intriguingly, miR-10b-5p, miR-342-3p, miR-186-5p, miR-361-3p, miR-155-3p, miR-130b-5p, and miR-744-5p also showed aberrant expression levels in neurodegenerative diseases, such as Alzheimer’s disease, MSA, and prion diseases^33^. These results demonstrated that high throughput sequencing-based detection and quantification of sncRNAs expression can provide more informative profiles that include all types and relative abundance of sncRNAs and their sequence variants, thereby significantly improving the overall performance of machine learning diagnostic classifiers and ultimately the AUC values of miRNA biomarkers^11,13^.

Notably, the predicted targets of miR-7-5p, miR-27b-3p, and miR-182-5p, were enriched in pathways such as ‘neuron projection’, ‘neurotransmitter secretion’, ‘generation of neurons’, and ‘nervous system development’ (Supplementary Fig. 4a). In addition, both miR-199a-5p and miR-182-5p were predicted to target the 3’UTR of several genes previously associated with parkinsonism (Supplementary Fig. 4b), as in the cases of ATP13A2 and SCARB2^4^.

In addition to the high specificity and long interval between iRBD onset and clinical manifestations of α-synucleinopathies, the prodromal phase of this disorder represents a unique opportunity for potential disease interventions^34^. To the best of our knowledge, this is the first EV-miRNA study in patients with iRBD with longitudinal follow-up. In our current study, miR-7-5p, miR-4665-5p, miR-5001-3p, miR-550b-3p were found to be correlated with RBD conversion. Our findings have implications for disease prediction strategies and early detection of synucleinopathies, such as PD and MSA, and further studies in other prodromal cohorts are warranted.

However, the current study also has several limitations. First, as a single-center study, it is uncertain whether these predictive markers are applicable to other populations with different exposures to environmental or genetic factors (i.e., ethnicity). Future work with larger cohorts from multiple centers is required to confirm these results, ideally through a prospective validation study. Second, only the miRNA signature was used in this study. A combination of miRNAs with v-PSG, neuroimaging, and neuropathological assessment may improve the sensitivity and specificity of diagnosis for prodromal PD (i.e., iRBD) and PD. Third, no available genetic data exists for the patients and healthy controls in the current cohort, which could help in the future to reduce the diagnostic bias for PD and iRBD. Additionally, we acknowledge the importance of replicating these findings in an independent cohort.

In conclusion, we established a cDNA library construction method, named EVsmall-seq, for high throughput sequencing of sncRNAs in plasma-EV using as low as 0.5 ng of total RNA input and identified miRNA signature features that could serve as biomarkers for distinguishing iRBD and/or PD from healthy individuals with high sensitivity and accuracy. Moreover, our study provides a valuable resource for sncRNA profiles in plasma EVs from the plasma of iRBD and PD patients and reveals an effective and non-invasive diagnostic strategy, with relevant biomarkers, for neurodegenerative diseases.

## Methods

### Study population and clinical assessment

A total of 169 participants were enrolled in this study between January 2019 and March 2020, and divided into three groups: 60 healthy individuals, 56 patients with iRBD, and 53 patients with PD. The healthy individuals were community volunteers from the same place with PD and iRBD patients in Shanghai between January 2019 and March 2020 without neurodegenerative disorders. All participants belonged to the Han Chinese ethnicity and were enrolled in Shanghai with no family history. The diagnosis of PD was performed according to the International Parkinson and Movement Disorder Society (MDS) diagnostic criteria by at least two neurologists skilled in movement disorders^35^, and all of the patients were diagnosed as idiopathic PD. The diagnosis of iRBD was made based on video-polysomnography evidence according to the standard International Classification of Sleep Disorders-III criteria (ICSD III). All iRBD patients were examined by neurologists to exclude those with motor signs of parkinsonism or secondary causes. Prospective follow-up was performed for these 56 iRBD patients for average 3.3 years. Diagnosis of parkinsonism was made according to current clinical diagnostic criteria by at least two movement disorders specialists. This study was approved by the ethics committee of Ruijin Hospital affiliated with the Shanghai JiaoTong University School of Medicine and was carried out at the Department of Neurology and Institute of Neurology of Ruijin Hospital. All participants or their guardians provided written informed consent.

For iRBD and PD patients, essential demographic, and clinical information, including a study questionnaire for motor and nonmotor manifestations of their disease, was collected and documented. The motor subscale of the Unified PD Rating Scale (UPDRS) was used to evaluate motor symptoms and ON medications. iRBD symptoms and their severity were evaluated by the REM Sleep Behavior Disorder Screening Questionnaire (RBDSQ). Nonmotor symptoms and autonomic dysfunction were evaluated by the Non-Motor Symptom Questionnaire (NMSQ) and Scale for Outcomes in PD-Autonomic (SCOPA-AUT), respectively. Depressive state was measured using the 17-item Hamilton Depression Rating Scale (HAMD); the Sniffin’ Sticks 16-item test (SS-16) was performed to assess olfactory function.

### Plasma sample collection and EV-RNA extraction

Plasma samples (0.5-1 ml) were collected from subjects in the fasting state. For each blood collection, 2 ml of venous blood was collected into 10-ml BD K_2_EDTA Vacutainer tubes (BD; Cat# 367525) to prevent coagulation. The anticoagulant-treated blood samples were immediately inverted several times and transferred to 2-ml conical tubes (Eppendorf; Cat# 0030120094). To obtain plasma, blood samples were centrifuged at 1300 × *g* for 10 min at room temperature (RT). The upper layer containing plasma was transferred to new tubes and centrifuged twice at 2500 × *g* for 15 min at RT to obtain platelet-poor plasma (PPP). Each plasma supernatant was carefully transferred to fresh 1-ml tubes and then stored at −80 °C until further use. Frozen plasma samples were thawed in a thermostat water bath for 2 min at 37 °C and then centrifuged at 2500 × *g* for 15 min at 4 °C to remove precipitated proteins and lipids. Approximately 0.5-1 ml of plasma was used as the starting material for EV isolation with an exoRNeasy Midi kit (Qiagen; Cat# 77144) according to the manufacturer’s instructions.

To ensure obtaining high-quality plasma EVs from patients, we established a procedure for plasma sample collection and protocols for EV isolation (Supplementary Fig. 5a). The morphology and size distribution of EVs were evaluated by transmission electron microscope (TEM) and nanoparticle tracking analysis (NTA). The size of isolated EVs ranged from 30 - 500 nm with a median diameter of 105 nm (Supplementary Fig. 5b, c). Furthermore, enrichment for the EV markers Alix and CD63 was observed in the EVs isolated from the plasma samples. In contrast, Calnexin and GRP94 were undetectable in isolated EVs, which indicated the absence of contamination by endoplasmic reticulum proteins (Supplementary Fig. 5d). All blots were processed in parallel and derived from the same experiments (Supplementary Fig. 6). On average, 2 nanograms (ng) of EV RNA was obtained from 0.5 milliliters (ml) of plasma with a range between 0.61 and 2.93 ng, and showed normal size distribution (Supplementary Fig. 5e) consistent with the previous study^20^. Approximately 0.5 ng of EV-RNA that passed the quantity and quality control criteria was used for small-RNA library construction and sequencing analyses. Since the sgRNA-guided Cas9 nuclease is capable of cleaving double-stranded DNA bearing a protospacer adjacent motif (PAM) sequence both in vitro and in vivo, we designed sgRNAs and introduced a Cas9/sgRNA in vitro cleavage step to reduce both the ysRNAs (Supplementary Fig. 5f) and the adapter dimers (Supplementary Data 12).

### EV isolation

For EV-RNA extraction, 1 ml of TRIzol reagent (Invitrogen; Cat# 15596026) was added to the column, which was centrifuged at 3000 × *g* for 1 min. Total EV-RNA was isolated by TRIzol reagent according to the manufacturer’s recommendations. The RNA pellet was dissolved in 11 μl of deionized water treated with diethylpyrocarbonate (DEPC); 1 μl was used for yield measurement of EV-RNA by Quant-iT RiboGreen RNA Assay Kit (Thermo Fisher Scientific; Cat# R11490); 2 μl was used for quality and size distribution examination by a 2200 Bioanalyzer (Agilent Technologies, CA, USA). The remaining 8 μl was used for sncRNA library construction and sequencing. For EV characterization, 1 ml of XE buffer was added to the column and centrifuged at 3000 × *g* for 1 min. Eluates were concentrated by ultrafiltration using Amicon Ultra-0.5 ml Centrifugal Filters with a molecular weight cutoff of 100 kDa (Millipore; Cat# UFC5003). EV was resuspended in 40 μl of phosphate-buffered saline (PBS) and was ready for downstream applications, including transmission electron microscopy (TEM), nanoparticle tracking analysis (NTA), and western blotting.

### Construction of the cDNA libraries from plasma EV-RNA

EV-RNA samples obtained from the plasma of healthy controls, iRBD patients, and PD patients were profiled by EVsmall-seq. For each sample, 0.5 ng RNA per sample was used as input material for generating sncRNA libraries. Ligation reaction for 5’ and 3’ adapters was performed according to the procedure described previously with some modifications (manuscript in preparation). In brief, adapter-ligated RNA was mixed with ProtoScript II Reverse Transcriptase, RNase Inhibitor, and reaction Buffer (NEB; Cat# M0358), and incubated for 1 h at 44 °C. After PCR amplification of RT products, the sequence-specific sgRNAs were assembled with Cas9 to cleave self-ligating adapters and RNA contaminants in the libraries. The oligo sequences of 3’adapter, biotin-RT primer, 5’adapter, adapter-sgRNA, ysRNA-sgRNA (Y RNA-derived small non-coding RNA sgRNA), P5 primer, and P7 primer used for sncRNA library construction were listed in Supplementary Data 12. Size selection of PCR products was performed by high-resolution polyacrylamide gel electrophoresis through 6% gels. For miRNAs, the bands corresponding to 135–145 bp were purified for sequencing. All gels derived from the same experiment and that they were processed in parallel.

### Transmission electron microscopy (TEM) analysis

Purified concentrated EVs in PBS were mixed with an equal volume of 4% paraformaldehyde (PFA). Five microliters of resuspended pellets were added to a formvar-carbon-coated EM grid and absorbed for 20 min in a dry environment. The grids were washed once with PBS for 1 min, followed by once with 1% glutaraldehyde for 5 min, and seven times with Milli-Q water for 2 min per wash. The grids were then negatively stained with 2.5% uranyl-oxalate solution for 10 min and air-dried for 5 min under incandescent light. Images were acquired on a Tecnai G2 Spirit TEM (FEI, The Netherlands) with a wide-angle AMT 2k CCD camera operating at 120 kV at the Shanghai Institute of Biochemistry and Cell Biology, Chinese Academy of Sciences.

### Nanoparticle tracking analysis (NTA)

The particle size and concentration of purified EV in PBS were analyzed using a ZetaView PMX 110 (Particle Metrix, Germany) equipped with a 405-nm laser and a high-sensitivity sCMOS camera. The ZetaView system was calibrated using 110 nm polystyrene particles. All measurements were performed at RT. Furthermore, NTA measurement was recorded and analyzed at 11 positions for 60 seconds at a frame rate of 30 frames/second. The result for each sample is presented as an average of the 3 measurements. Particle movement was analyzed using NTA software (ZetaView 8.04.02 SP2).

### Western blotting

For protein analysis, the protein concentrations of purified EV in PBS were quantified using a bicinchoninic acid (BCA) assay (Beyotime; Cat# P0010S). Ten micrograms of total protein of each sample were denatured in 4× sodium dodecyl sulfonate (SDS) buffer at 95 °C for 5 min before being separated through 8% polyacrylamide SDS gels and transferred to nitrocellulose membranes. The membranes were blocked with 5% (wt/vol) fat-free milk in PBS with Tween (PBST) for 1 h at RT prior to incubation with anti-Alix antibody (1:1000 dilution; Abcam; Cat# 186429), anti-CD63 antibody (1:200 dilution; Abcam; Cat# 193349), anti-Calnexin antibody (1:1000 dilution; Abcam; Cat# 22595), and anti-GRP94 antibody (1:500 dilution; Abcam; Cat# 108606). All primary antibodies were diluted in 5% fat-free milk, and blots were probed overnight at 4 °C. The membranes were washed four times for 10 min and incubated with HRP-conjugated secondary antibodies for 1 h at RT. The secondary antibodies were goat anti-rabbit IgG for Alix, GRP94, or Calnexin (1:5000 dilution; MultiSciences; Cat# GAR007) and goat anti-mouse IgG for CD63 (1:5000 dilution; MultiSciences; Cat# GAM007). The protein bands were detected with a Pierce ECL western blotting substrate kit (Thermo Fisher Scientific; Cat# R32209).

### Sources for sequences and genome assemblies

The genomic sequences of human (hg38) were downloaded from the University of California Santa Cruz (UCSC) Genome Browser^36^ (http://genome.ucsc.edu/). Known RNA sequences were retrieved from the following databases: miRNA, miRbase (version 22.0)^37^; tRNAs, Genomic tRNA Database (http://lowelab.ucsc.edu/GtRNAdb); Y RNAs, rRNAs, 5.8 S, 18 S, 28 S and 45 S from NCBI GenBank (http://www.ncbi.nlm.nih.gov/), 5 S from Ensembl (http://www.ensembl.org/index.html); and snoRNAs, lncRNAs and mRNAs from Ensembl (http://www.ensembl.org/index.html).

### High throughput sequencing and data analyses of sncRNAs

All the cDNA libraries of sncRNAs were sequenced on NovaSeq (Illumina). We used cutadapt to clip adapters and filter out low-quality reads^38^. Reads failing to match the adaptor or reads with lengths shorter than 17 nt were discarded. Redundant sequences were collapsed as useful reads for further analysis^39^. Then, we aligned the reads to reference sequences by bowtie^40^. The reads that matched the 5’ start site of annotated miRNAs and matched the 3’ ends with at most 3 nt deletions and/or 3 nt additional sequences derived from pre-miRNAs were counted in the abundance of miRNAs. The miRNA expression level was normalized by size factor^41^. SncRNAs with more than one annotation were characterized in the following order: miRNA, ysRNA, tsRNA (tRNA-derived small non-coding RNA), rsRNA (rRNA-derived small non-coding RNA), snRNA, snoRNA, lncRNA, and mRNA. Sequences that were not annotated with any of the RNA categories above were classified as others. The miRNA expression profiles in human tissues were downloaded from TissueAtlas^42^. The gene ontology enrichment analysis of miRNA’ target genes was performed by miRPathDB^43^. miRNA’s target sites for corresponding genes were predicted by TargetScan^44^. The miRNA expression patterns from healthy to iRBD to PD samples were identified by R package Mfuzz^45^.

### Statistical analysis

We used the R software package for statistical analysis. Batch correction was conducted by limma^46^. When differential expression analysis and classification were performed between PD and Healthy or PD and iRBD groups, limma was used to correct for gender bias^46^. Differentially expressed miRNAs were calculated by limma^46^ (*p* value < 0.05, fold change>1.4). The adjusted *p* value was calculated based on Benjamini-Hochberg (BH) correction. We used the prcomp package for principal component analysis (PCA), which was visualized by ggplot2^47^. Unsupervised hierarchical clustering analysis was conducted and visualized from pheatmap^48^. The comparisons between two groups were performed with a Wilcoxon rank sum test, whereas comparisons among three or more groups were performed with a Kruskal-Wallis rank sum test. The statistical tests were performed and visualized by ggplot2 and ggpurb^49^.

Features were selected with the R package Boruta to find all relevant variables for machine learning^50^. We used the differentially expressed miRNAs as input for Boruta feature selection. We used a 5-fold cross-validation on the training set to assess the accuracy rate for classification^51^. The diagnostic efficacy was evaluated by receiver operating characteristic (ROC) curve analysis for the training and validation cohorts^52^. The comparison between areas under the curve (AUCs) of different classifiers was evaluated by the bootstrap method with 100 iterations. For longitudinal data, the endpoint was defined as the time from baseline RBD to the conversion of PD/MSA. For miRNA predictor selection, univariate Cox proportional hazards regression analysis was repeated to identify miRNAs associated with RBD conversion, and miRNAs with significant *P* values (*p* < 0.05) were selected as candidates for Kaplan-Meier analysis. We used R survival and surviminer packages to determine the best cut-off values for dividing the patients into high- and low-level miRNA expression groups and plotted Kaplan-Meier curves.

### Reporting Summary

Further information on research design is available in the Nature Research Reporting Summary linked to this article.

### Supplementary information

Supplemental information

nr-reporting-summary1

Supplementary Data

## Data Availability

The deep sequencing data have been deposited in the National Center for Biotechnology Information Gene Expression Omnibus under accession number GSE166070.

## Code availability

The code of bioinformatic analysis and processed data used during the current study have been deposited to the GitHub repository (https://github.com/liuwell/PDbiomarker).
