# Supplementary material for: Candidate biomarkers of EV-microRNA in detecting REM sleep behavior disorder and Parkinson’s disease
Source: NPJ Parkinsons Dis. 2024 Jan 10;10:18. doi: 10.1038/s41531-023-00628-4 (PMC10781790; doi:10.1038/s41531-023-00628-4)
Supplement: Supplementary file 1 — Supplemental information [file 41531_2023_628_MOESM1_ESM.pdf]

Supplementary Information

Supplementary Table 1. Baseline and clinical characteristics of training and validation sets

Training set				
Clinical parameters	Healthy	iRBD	PD	*P* value
No. of participants	35	34	32	/
Age	61.3 ± 9.3	63.2 ± 7.8	62.8 ± 9.2	0.80
Sex (M/F)	18/17	20/14	16/16	0.77
UPDRS III	/	/	30.5 ± 18.7	/
RBDSQ	/	8.3 ± 3.7	5.1 ± 3.3	<0.01
NMSQ	/	7.6 ± 4.2	8.2 ± 3.0	0.57
SS-16	/	9.0 ± 4.8	7.2 ± 3.2	0.10
HAMD	/	3.9 ± 3.9	5.0 ± 4.5	0.38
SCOPA-AUT	/	8.7 ± 5.3	15.9 ± 16.0	0.07
**Validation set**				
Clinical parameters	Healthy	iRBD	PD	*P* value
No. of participants	25	22	21	/
Age	63.2 ± 10.2	65.2 ± 6.6	63.3 ± 8.9	0.71
Sex (M/F)	16/9	14/8	9/12	0.27
UPDRS III	/	/	23.0 ± 15.1	/
RBDSQ	/	7.9 ± 2.3	4.8 ± 3.2	<0.05
NMSQ	/	6.0 ± 3.3	8.7 ± 5.3	0.19
SS-16	/	8.5 ± 3.4	8.0 ± 3.1	0.70
HAMD	/	3.3 ± 4.1	9.2 ± 7.3	0.05
SCOPA-AUT	/	6.6 ± 3.3	11.8 ± 12.1	0.21

iRBD: idiopathic rapid eye movement sleep behavior disorder; PD: Parkinson's disease; UPDRS III: the Unified PD Rating Scale III; RBDSQ: the REM Sleep Behavior Disorder Screening Questionnaire; NMSQ: Non-Motor Symptom Questionnaire; SS-16: the Sniffin' Sticks 16-item test; HAMD: the 17-item Hamilton Depression Rating Scale; SCOPA-AUT: Scale for Outcomes in PD-Autonomic.

Supplementary Table 2. miRNAs associated with iRBD conversion.

miRNAs	Cut-off values	HR	95% CI	P value
miR-7-5p	325.64	1.0059	1.0001-1.0117	0.0469
miR-4665-5p	64.23	1.0122	1.0029-1.0216	0.0103
miR-5001-3p	13.93	1.0134	1.0029-1.024	0.0122
miR-550b-3p	22.12	1.0346	1.0107-1.0591	0.0043

All results were adjusted for age and sex. HR: hazard ratio; CI: confidence intervals.

Supplementary Figure 1. Differentially expressed EV-associated miRNAs between iRBD and PD-iRBD or PD-iRBD and PD-no-iRBD

a. MA-plot of differentially expressed miRNAs between PD-iRBD patients and iRBD patients. The up- or down-regulated miRNAs are shown in red or blue, respectively. **b.** Unsupervised clustering heatmap of differentially expressed miRNAs of PD-iRBD patients (blue) and iRBD patients (red). Each row represents a differentially expressed miRNA. **c.** MA-plot of the differentially expressed miRNAs between PD-no-iRBD patients and PD-iRBD patients. The up- or down-regulated miRNAs are shown in red or blue, respectively. **d.** Unsupervised clustering heatmap of differentially expressed miRNAs of PD-no-iRBD patients (blue) and PD-iRBD patients (red). Each row represents a differentially expressed miRNA. **e.** Boxplot of differentially expressed miRNAs between iRBD and PD subgroups. PD-iRBD: PD patients who also have iRBD; PD-no-iRBD: PD patients without iRBD. The estimated *P*-values are based on the Wilcoxon rank sum test and are shown in the boxplot. * : P value < 0.05, ** : P value < 0.01, *** : P value < 0.001, n.s. : no significance. The lower and upper hinges correspond to the first and third quartiles (the 25th and 75th percentiles). The center line correspond to the median. The upper whisker extends from the hinge to the largest value no further than 1.5 * IQR from the hinge (where IQR is the inter-quartile range, or distance between the first and third quartiles). The lower whisker extends from the hinge to the smallest value at most 1.5 * IQR of the hinge. Data beyond the end of the whiskers are called "outlying" points and are plotted individually.

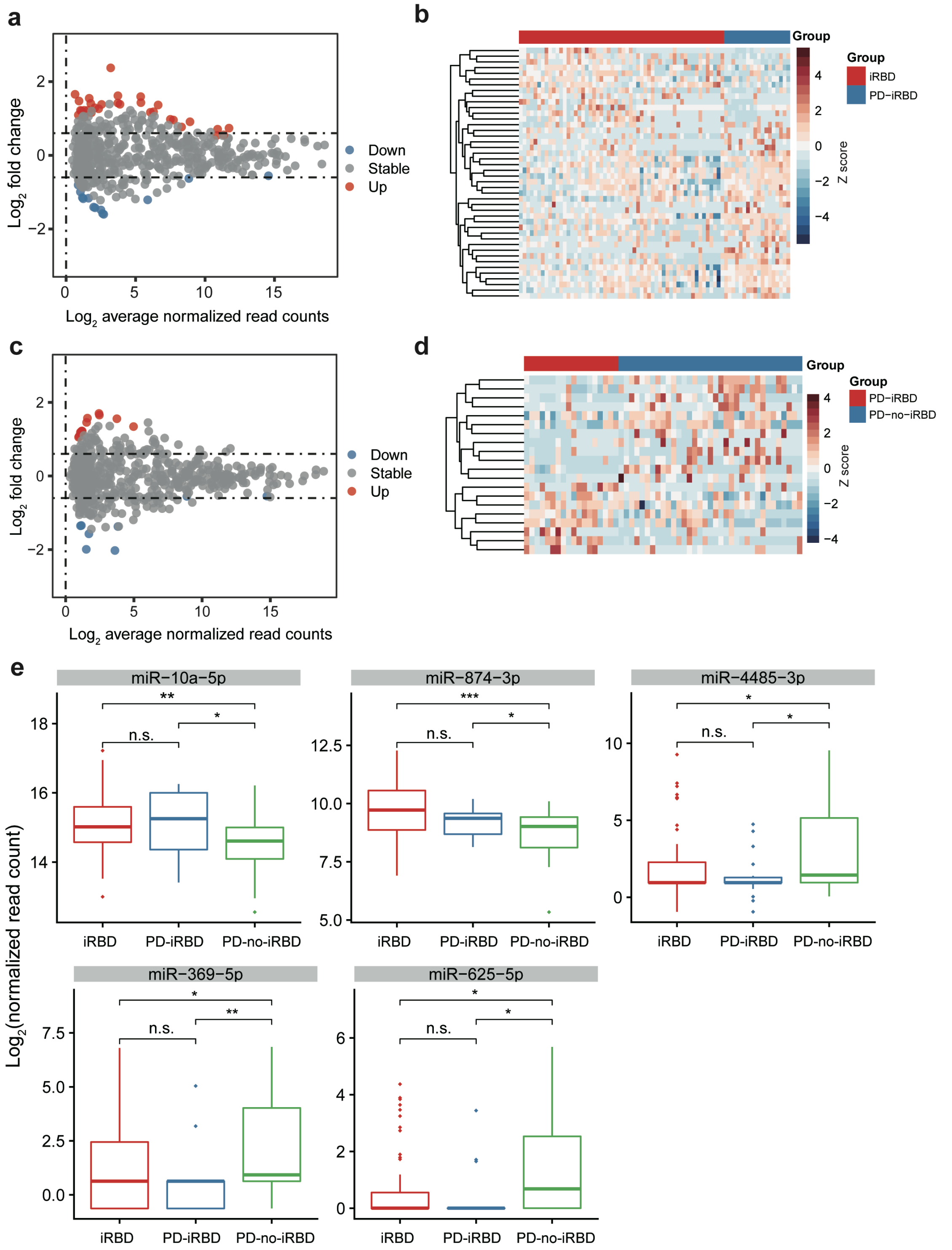
a
b
c
d
e
Log2 fold change
Log2 average normalized read counts
Down
Stable
Up
Group
iRBD
PD−iRBD
PD−no−iRBD
Z score
miR−10a−5p
miR−874−3p
miR−4485−3p
miR−369−5p
miR−625−5p
Log2(normalized read count)
iRBD
PD-iRBD
PD-no-iRBD
n.s.

Supplementary Figure 2. EV-associated sncRNAs library construction

a. EVsmall-seq RNA (yellow) was sequentially ligated to 3' (blue) and 5' (red) adapters. The ligation products were reverse-transcribed using a biotin-labelled reverse transcription (RT) primer (purple), followed by Cas9/single guide RNA (sgRNA) cleavage to eliminate adapter heterodimers and ysRNA fragments. After two rounds of PCR amplification to suppress bias during PCR exponential amplification, the library was size selected using polyacrylamide gel electrophoresis (PAGE) for high-throughput sequencing. **b.** Comparison of the miRNA bands generated from 0.5 to 2 nanograms (ng) of EV RNA from human plasma. The miRNA band was approximately 140 bp long and detected using 6% PAGE. **c.** Comparison of cDNA library construction with or without Cas9/sgRNA treatment. For each plasma sample, 0.5 ng of total EV-RNA was used as input. PCR amplification products were analyzed using 6% PAGE. **d.** A boxplot of the proportion of ysRNAs among the total mapped reads from the cDNA library with (blue) or without (red) Cas9/sgRNA treatment. Each group contained four biological replicates. **e.** Representative scatter plot of miRNA expression in the cDNA library with (y-axis) or without (x-axis) Cas9/sgRNA treatment. Each dot represents one miRNA species. The adjusted R-squared value of the linear regression is shown in blue. **f.** Spearman correlation heatmap of miRNA expression in the cDNA libraries treated with or without Cas9/sgRNA. The correlation coefficient of four groups of paired samples is shown in the plot. Ctrl: without Cas9/sgRNA treatment; Cas9: Cas9/sgRNA treatment.

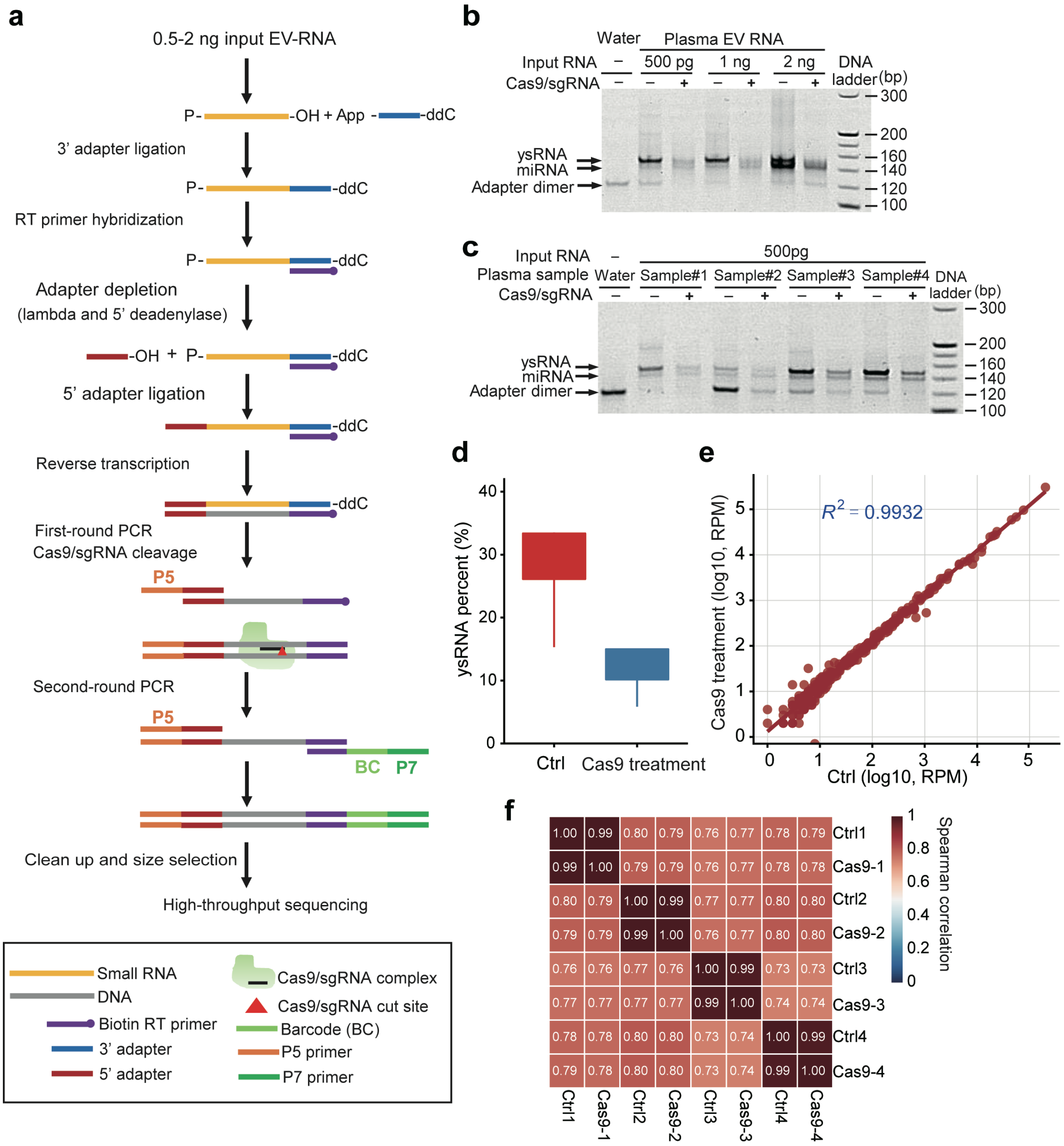
a
0.5-2 ng input EV-RNA
P- -OH + App - -ddC
3' adapter ligation
P- -ddC
RT primer hybridization
P- -ddC
Adapter depletion
(lambda and 5' deadenylase)
-OH + P- -ddC
5' adapter ligation
-ddC
Reverse transcription
-ddC
First-round PCR
Cas9/sgRNA cleavage
P5
Second-round PCR
P5
BC P7
Clean up and size selection
High-throughput sequencing
Small RNA
DNA
Biotin RT primer
3' adapter
5' adapter
Cas9/sgRNA complex
Cas9/sgRNA cut site
Barcode (BC)
P5 primer
P7 primer
b
Water
Plasma EV RNA
Input RNA – 500 pg 1 ng 2 ng
Cas9/sgRNA – – + – + – +
DNA ladder (bp)
300 200 160 140 120 100
ysRNA
miRNA
Adapter dimer
c
Input RNA – 500pg
Plasma sample Water Sample#1 Sample#2 Sample#3 Sample#4
Cas9/sgRNA – – + – + – + – +
DNA ladder (bp)
300 200 160 140 120 100
ysRNA
miRNA
Adapter dimer
d
ysRNA percent (%)
0 10 20 30 40
Ctrl
Cas9 treatment
e
R^2 = 0.9932
Cas9 treatment (log10, RPM)
Ctrl (log10, RPM)
0 1 2 3 4 5
f
Ctrl1 1.00 0.99 0.80 0.79 0.76 0.77 0.78 0.79
Cas9-1 0.99 1.00 0.79 0.79 0.76 0.77 0.78 0.78
Ctrl2 0.80 0.79 1.00 0.99 0.77 0.77 0.80 0.80
Cas9-2 0.79 0.79 0.99 1.00 0.76 0.77 0.80 0.80
Ctrl3 0.76 0.76 0.77 0.76 1.00 0.99 0.73 0.73
Cas9-3 0.77 0.77 0.77 0.77 0.99 1.00 0.74 0.74
Ctrl4 0.78 0.78 0.80 0.80 0.73 0.74 1.00 0.99
Cas9-4 0.79 0.78 0.80 0.80 0.73 0.74 0.99 1.00
Ctrl1 Cas9-1 Ctrl2 Cas9-2 Ctrl3 Cas9-3 Ctrl4 Cas9-4
Spearman correlation
1 0.8 0.6 0.4 0.2 0

Supplementary Figure 3. Profile of plasma EV-associated sncRNAs using high throughput sequencing

a. The sequencing depth of sncRNAs in healthy, iRBD, and PD samples. M: one million reads. The mean and standard deviation of sequencing depth is shown in black. **b, c.** The percentage of high-quality reads (b) or mapped reads (c) in healthy, iRBD, and PD samples. **d, e.** The average proportion (d) and length distribution (e) of various types of sncRNAs in plasma-derived EVs. **f.** The number of detected miRNA species in plasma EVs.

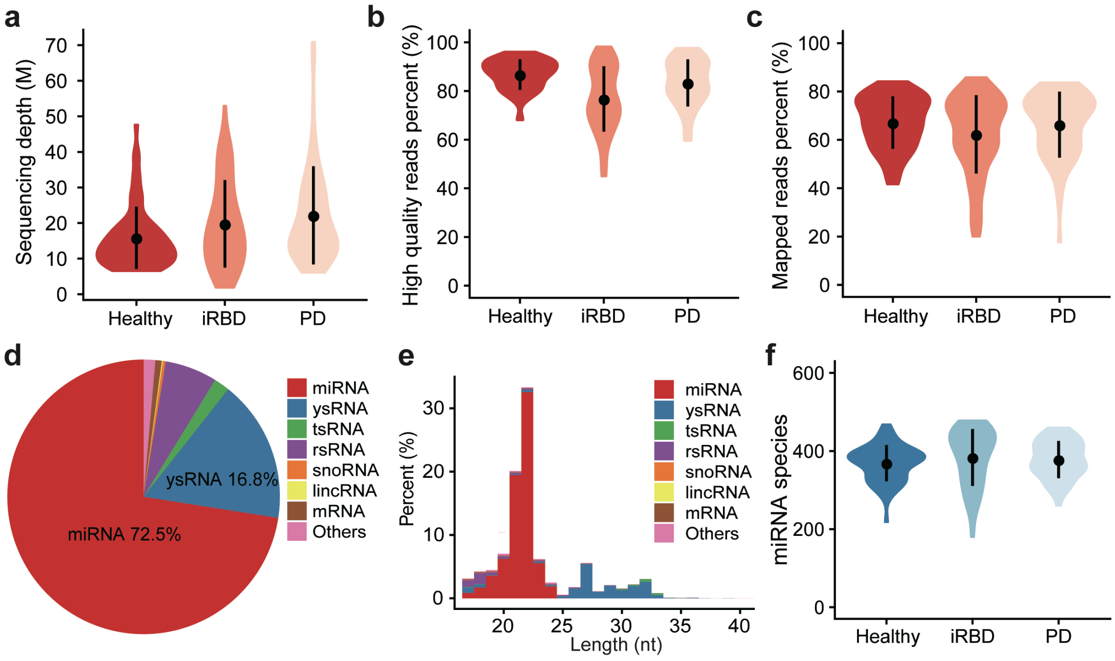

Supplementary Figure 4. The gene ontology enrichment analysis of miRNA targets and predicted miRNA target sites in the 3'UTR

a.The top 20 most significant pathways of targets are shown in the bar plot for miR-7-5p, miR-27b-30, and miR-182-5p. The pathways associated with neurons are highlighted in red**. b.**The miR-199a-5p target site in the 3'UTR of ATP13A2. The miR-182-5p target sites in the 3'UTR of SCARB2. The predicted base-pairings between miRNAs and the target sequences are shown.

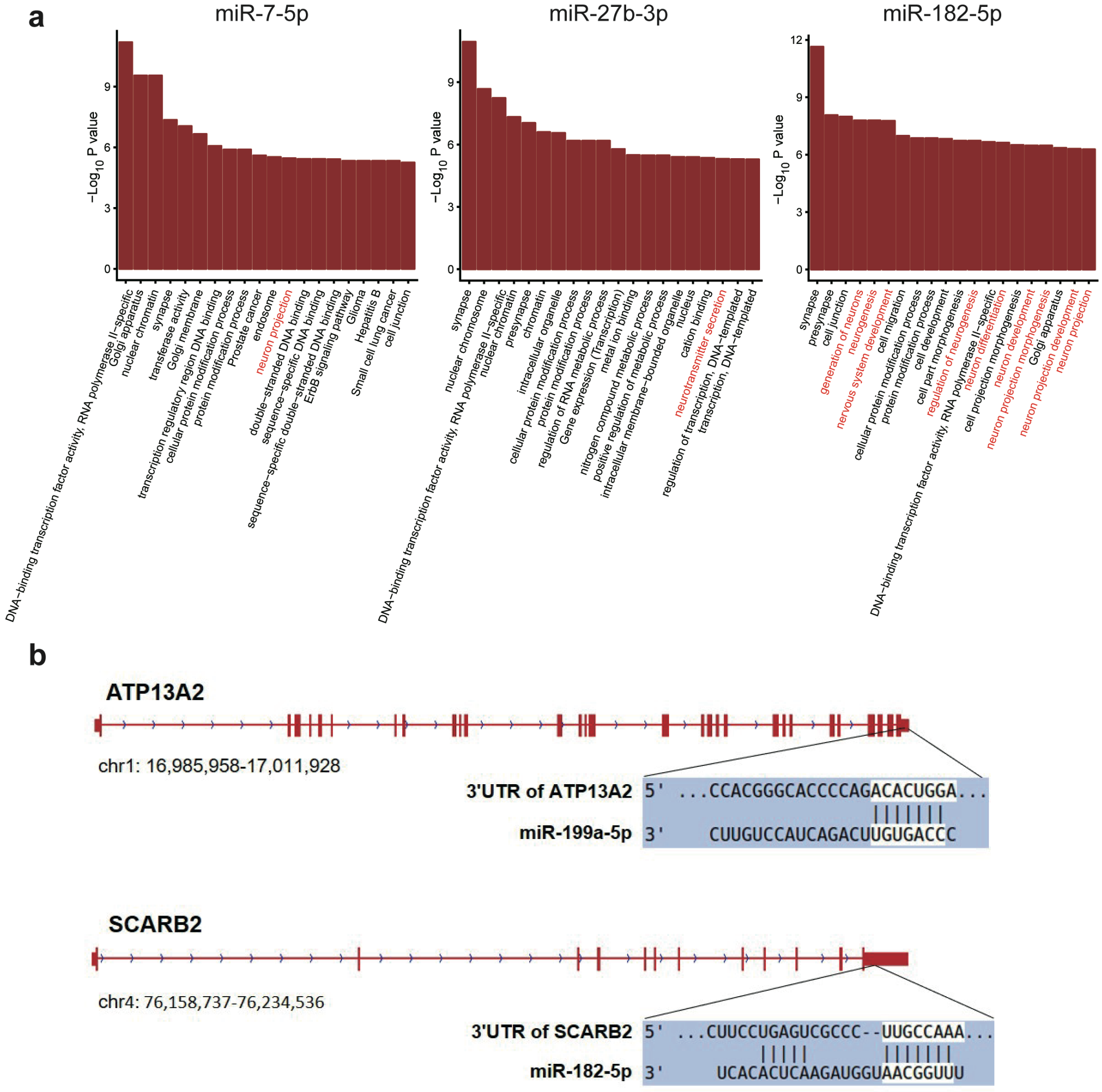

Supplementary Figure 5. Characterization of Extracellular Vehicles (EVs) isolated from plasma

a. Diagram of the workflow for isolation and analysis of sncRNAs associated with plasma EVs. **b.** Representative graph of the nanoparticle tracking analysis (NTA) showing the concentration and size (diameter) distribution of EVs isolated from plasma samples. **c.** Representative transmission electron microscope (TEM) image of isolated EVs. **d.** Western blot detection of EVs using antibodies against Alix and CD63 in isolated EVs from plasma samples and peripheral blood mononuclear cell (PBMC) lysates (negative control). Calnexin and GRP94 were absent in the EV samples and served as negative controls. **e.** A standard EV-RNA size distribution map analyzed by 2200 Bioanalyzer. There was a high major peak between 50 and 200 nucleotides (nt) and some low peaks between 200 and 500 nt. The amount above 500 nt was extremely low and almost nonexistent. **f.** Cas9/sgRNA design for specific cleavage of amplified products of ysRNA fragments in the cDNA library of plasma EV-associated sncRNAs. 5' adapter (purple); 3' adapter (green); ysRNA-by-product (blue); sequence targeted by sgRNA (yellow); PAM sequence (CGG, red).

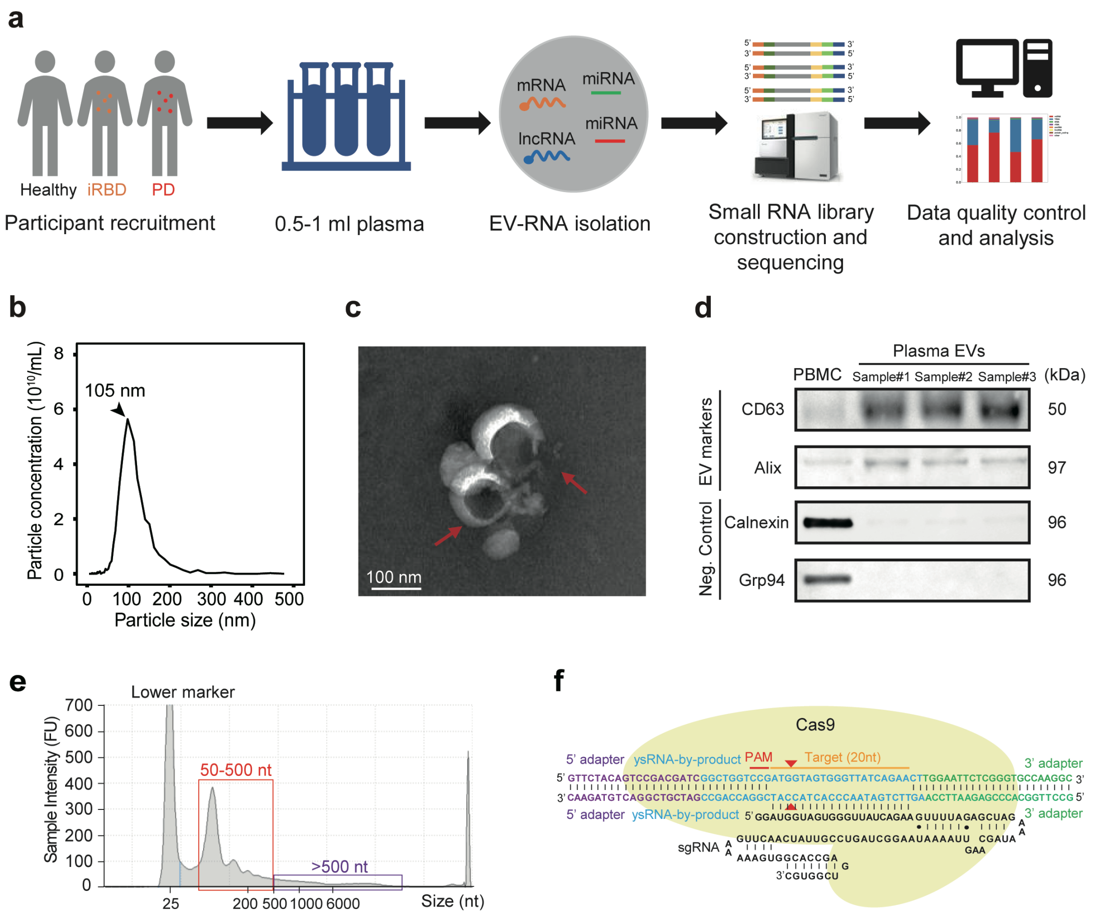

Supplementary Figure 6. The full and uncropped western blot

a, b. Western blot detection of EVs using antibodies against CD63 (a) and Alix (b) in isolated EVs from plasma samples and peripheral blood mononuclear cell (PBMC) lysates (negative control). **c, d.** Calnexin (c) and GRP94 (d) were absent in the EV samples and served as negative controls.

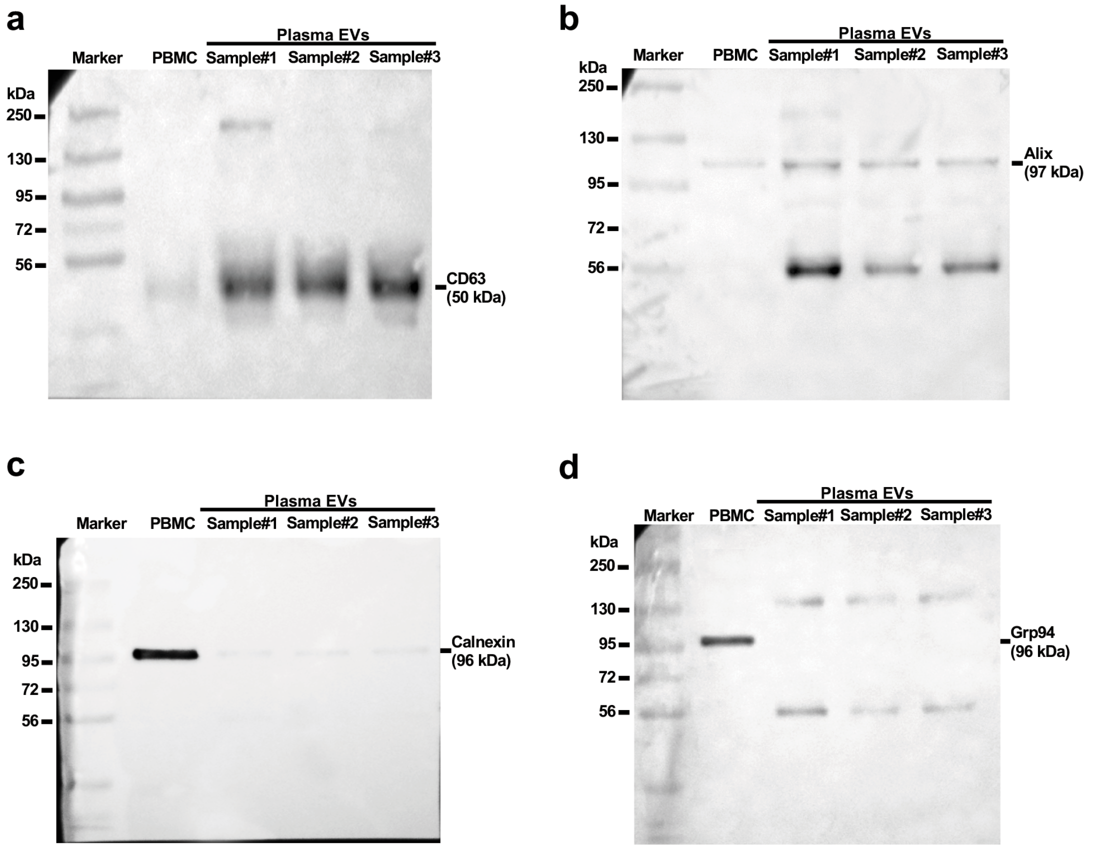

Supplementary Data 1. Differentially expressed miRNAs in PD patients compared with healthy individuals.

Supplementary Data 2. The miRNA biomarkers for distinguishing PD patients.

Supplementary Data 3. Differentially expressed miRNAs in iRBD patients compared with healthy individuals.

Supplementary Data 4. The miRNA biomarkers for distinguishing iRBD patients.

Supplementary Data 5. Differentially expressed miRNAs in PD patients compared with iRBD patients.

Supplementary Data 6. miRNA biomarkers for distinguishing PD from iRBD patients.

Supplementary Data 7. Differentially expressed miRNAs in PD-iRBD patients compared with iRBD patients.

Supplementary Data 8. Differentially expressed miRNAs in PD-no-iRBD patients compared with PD-iRBD patients.

Supplementary Data 9. List of miRNAs with different expression patterns from healthy controls to iRBD to PD samples.

Supplementary Data 10. List of sncRNA sequencing information (including sequencing depth, batch information, high quality reads ratio, mapped reads ratio, and the number of detected miRNA species).

Supplementary Data 11. miRNA expression matrix for all samples.

Supplementary Data 12. The summary of oligo sequences.
